# Radiative transfer modelling reveals why canopy reflectance follows function

**DOI:** 10.1038/s41598-019-43011-1

**Published:** 2019-04-25

**Authors:** Teja Kattenborn, Sebastian Schmidtlein

**Affiliations:** Institute of Geography and Geoecology (IFGG), Karlsruhe Institute of Technology (KIT), Kaiserstr. 12, 76131 Karlsruhe, Germany

**Keywords:** Physiology, Ecology

## Abstract

Optical remote sensing is potentially highly informative to track Earth’s plant functional diversity. Yet, causal explanations of how and why plant functioning is expressed in canopy reflectance remain limited. Variation in canopy reflectance can be described by radiative transfer models (here PROSAIL) that incorporate plant traits affecting light transmission in canopies. To establish causal links between canopy reflectance and plant functioning, we investigate how two plant functional schemes, i.e. the Leaf Economic Spectrum (LES) and CSR plant strategies, are related to traits with relevance to reflectance. These traits indeed related to both functional schemes, whereas only traits describing leaf properties correlated with the LES. In contrast, traits related to canopy structure showed no correlation to the LES, but to CSR strategies, as the latter integrates both plant economics and size traits, rather than solely leaf economics. Multiple optically relevant traits featured comparable or higher correspondence to the CSR space than those traits originally used to allocate CSR scores. This evidences that plant functions and strategies are directly expressed in reflectance and entails that canopy ‘reflectance follows function’. This opens up new possibilities to understand differences in plant functioning and to harness optical remote sensing data for monitoring Earth´s functional diversity.

## Introduction

Through natural selection plants have diversified in various functions in order to adapt to environmental conditions, including abiotic factors (e.g. precipitation or nutrient gradients) and biotic interactions (e.g. competition or herbivory). Assessing patterns of plant functioning in space and time is a prerequisite to understand biosphere-atmosphere interactions and ecosystem dynamics such as community assembly or nutrient cycles^1–5^. With accelerated global change the data demand on patterns of plant functioning has increased, as the latter is heavily affected by anthropogenic impacts^6–9^. However, due to vast temporal and spatial variation in plant functions, and the complexity to retrieve the latter in an explicit, consistent and spatially exhaustive way, data of Earth’s plant functional diversity remain limited^5,10^. To close this gap optical Earth observation data is potentially highly informative^11,12^. During recent years, various studies have demonstrated that optical Earth observation data allows mapping variation in plant functioning, functional types and strategies^13–20^. However, it often remains unclear why we can remotely sense differences in plant functioning^11,21,22^. To fully harness the potential of Earth observation data and to improve available algorithms, it is crucial to understand the underlying processes that enable us to monitor plant functioning. The key to such understanding are the traits that contribute to canopy reflectance.

**Figure 1:**
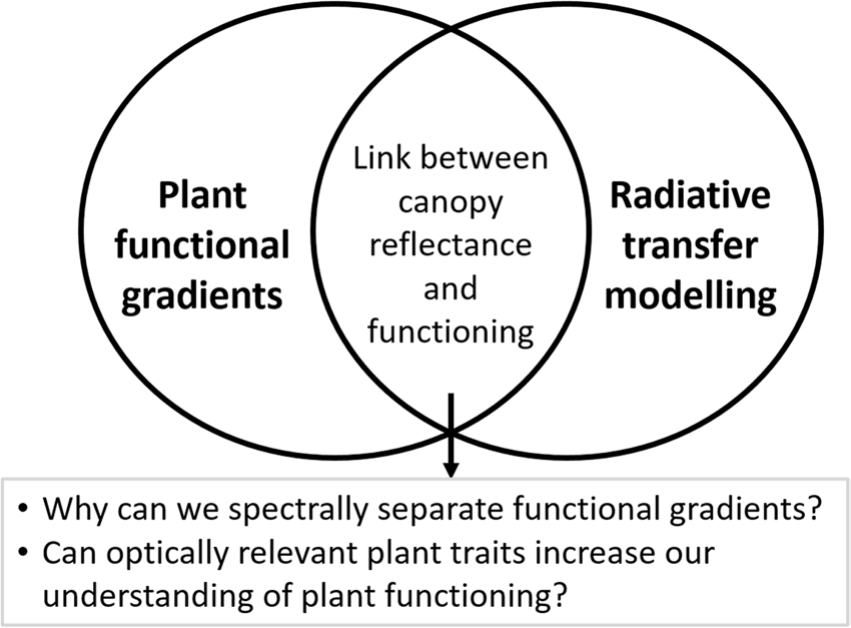
Rationale of linking plant functioning with radiative transfer modelling.

The mechanics of solar radiation in plant canopies, including light emitted from plant canopies and thus retrievable from Earth observation sensors, is already well understood and formulated in process-oriented models, i.e. canopy radiative transfer models (RTMs). Although radiative transfer is determined by traits with relevance for plant functioning, few studies have explicitly linked RTMs and plant functioning^19,23,24^. Such RTMs are particularly determined by canopy characteristics defining the amount of light being intercepted or scattered by the foliage as well as foliage properties (e.g. leaf constituents or structure) defining leaf internal scattering, absorption and transmission rates. Here, we assess the distribution of these traits along plant functional gradients, because knowing more about the links between optically relevant traits and plant functioning allows for mapping and monitoring plant functions in a more mechanistic way. This could dramatically improve the robustness and transferability of our models. Furthermore, it can be assumed that bridging plant functioning and canopy reflectance with radiative transfer theory can increase our understanding of how environmental factors and biotic interactions shape plant functional diversity^11^ (Fig. 1).

As optically relevant traits we addressed those traits that are incorporated in PROSAIL^25–27^, which is the most widely applied RTM for plant canopies. It couples two models, firstly PROSPECT modelling the leaf optical properties (e.g. pigment or water content), and secondly 4SAIL taking into account the structural properties of a canopy (e.g. leaf orientation) and its relative orientation to the sun and sensor^25^. We also considered traits that can be directly deduced from the original PROSAIL trait space (e.g. leaf pigments by leaf mass or fAPAR). A summary of traits and assumed links to plant functions is provided in Table 1.

**Table 1:** The optically relevant plant traits considered in the present study and their functions.

	Trait/Parameter [unit]	Abbr.	Description/functional role
Traits incorporated in PROSAIL-D	Chlorophyll content [μg/cm²]	Cab_area_	Leaf pigments chlorophyll a + b; primary molecule for light harvesting^27^
	Carotenoid content [μg/cm²]	Car_area_	Leaf pigments including xantophylls and carotenes; photoprotection and light harvesting^27^
	Anthocyanin content [μg/cm²]	Ant_area_	Leaf pigments of the flavonoid family; photoprotection, protection from pathogens^58,72^
	Leaf Area Index [m²/m²]	LAI	Ratio of total one sided leaf area per unit ground; dominant control of primary productivity and transpiration^24,52,73^
	Leaf Inclination Distribution Function [deg.]	LIDF	Variation of leaf angles in the canopy; controls light harvesting efficiency, leaf temperature and transpiration^40,74^
	Leaf Mass per Area [g/cm²]	LMA	Inverse of Specific Leaf Area (SLA), Aggregates leaf constituents such as sugar, starch, cellulose or lignin; well-known proxy for resource allocation and plant strategies^4,28^
	Equivalent Water Thickness [mg/cm²]	EWT	Water content per leaf area; determines thermal regulation, drought resistance and flammability^75–77^
	Mesophyll structure coefficient [-]	N_meso_	Artificially designed PROSPECT parameter, relating to the thickness of the mesophyll layer, which affects light harvesting and light transmission as well as CO_2_ diffusion^55,78^
	Brown pigment content [-]	Cbrown	Artificial PROSPECT parameter, relates to polyphenols such as tannins and other secondary metabolites with functions such as UV protection or defensive compounds against herbivory and pathogens^79,80^
Traits derived from PROSAIL-D trait space	Canopy Leaf Mass per Area [g/m²]	LMA_canopy_	Total leaf mass per canopy area [m²] calculated as the product of LAI and LMA
	Canopy water content [g]	EWT_canopy_	Total water content per canopy area [m²] calculated as the product of LAI and EWT
	Chlorophyll conc. [‰]	Cab_mass_	Chlorophyll mass per leaf dry mass
	Carotenoid conc. [‰]	Car_mass_	Carotenoid mass per leaf dry mass
	Anthocyanin conc. [‰]	Ant_mass_	Anthocyanin mass per leaf dry mass
	Fraction of Absorbed Photosynthetically Active Radiation [%]	fAPAR	Fraction of photosynthetic active radiation (PAR) absorbed in canopy. Integrates absorption by pigments and canopy structural traits (LAI, ALA); reflects gross photosynthetic capacity of the canopy. Simulated using PROSAIL (S3)
	Accumulated Absorbed PAR [kWh/m²]	APAR_cum_	PAR absorbed within the growing season; derived from multiplying fAPAR with course of direct and diffuse radiation during species specific growth length (S3)

These are traits implemented in PROSAIL and derivatives thereof.

We assessed the distribution of these traits across plant functional gradients using *in-situ* measurements acquired in an outdoor experimental setting with plants grown in pots. The species pool comprised 45 herbaceous species from Central Europe and covered a broad functional spectrum.

Instead of looking at individual functions such as carbon sequestration or evapotranspiration, we referred to more general expressions of plant functioning provided by two well-established schemes: the Leaf Economic Spectrum (LES^3^) and Grime’s CSR model of plant strategy types (CSR^28,29^). Both schemes are general approximations of principal functional differences between species that bundle many individual functions to metrics characterising their overall performance towards abiotic and biotic environmental selection pressures.

**Figure 2:**
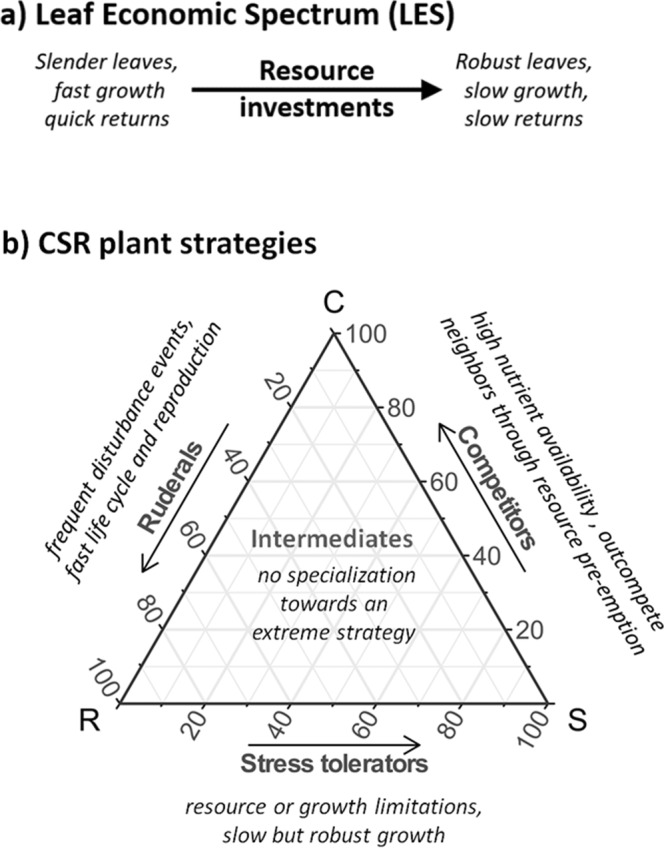
Schemes of plant functional gradients that were compared to optically relevant traits of cultivated plants: (**a**) Leaf Economic Spectrum (LES), and (**b**) CSR plant strategies.

The LES was derived from analysing various leaf traits (leaf lifespan, leaf mass per area, photosynthetic capacity, dark respiration rate, nitrogen, and phosphorus concentration) and describes the spectrum of leaf resource investments ranging from fast and acquisitive (low investment) to slow and conservative growth (high investment) (Fig. 2a)^3^. We compared optically relevant traits to the LES, as resource economics in leaves have been found to reflect a main axis of functional differences in plants^3,30^ and can thus assumed to be directly linked to the optical leaf traits in PROSAIL. The CSR model characterises plant species by means of three axes, defining their competitive (C), stress tolerating (S) and ruderal abilities (R) (Fig. 2b)^28,29^. Competitors are adapted to nutrient-rich sites featuring rapid growth to preempt resources. Stress-tolerators compensate environmental conditions limiting metabolic function through slow and robust growth, whereas ruderals are small-sized with short life cycles to counteract lethal events of disturbance or biomass removal. We compared optically relevant traits to the CSR scheme as it, in contrast to the LES, further integrates differences in function at the whole plant level^29,31^ and, thus, may be more appropriate for assessing optically relevant traits that are related to the canopy structure.

## Results

### Optically relevant traits versus Leaf Economic Spectrum

**Figure 3:**
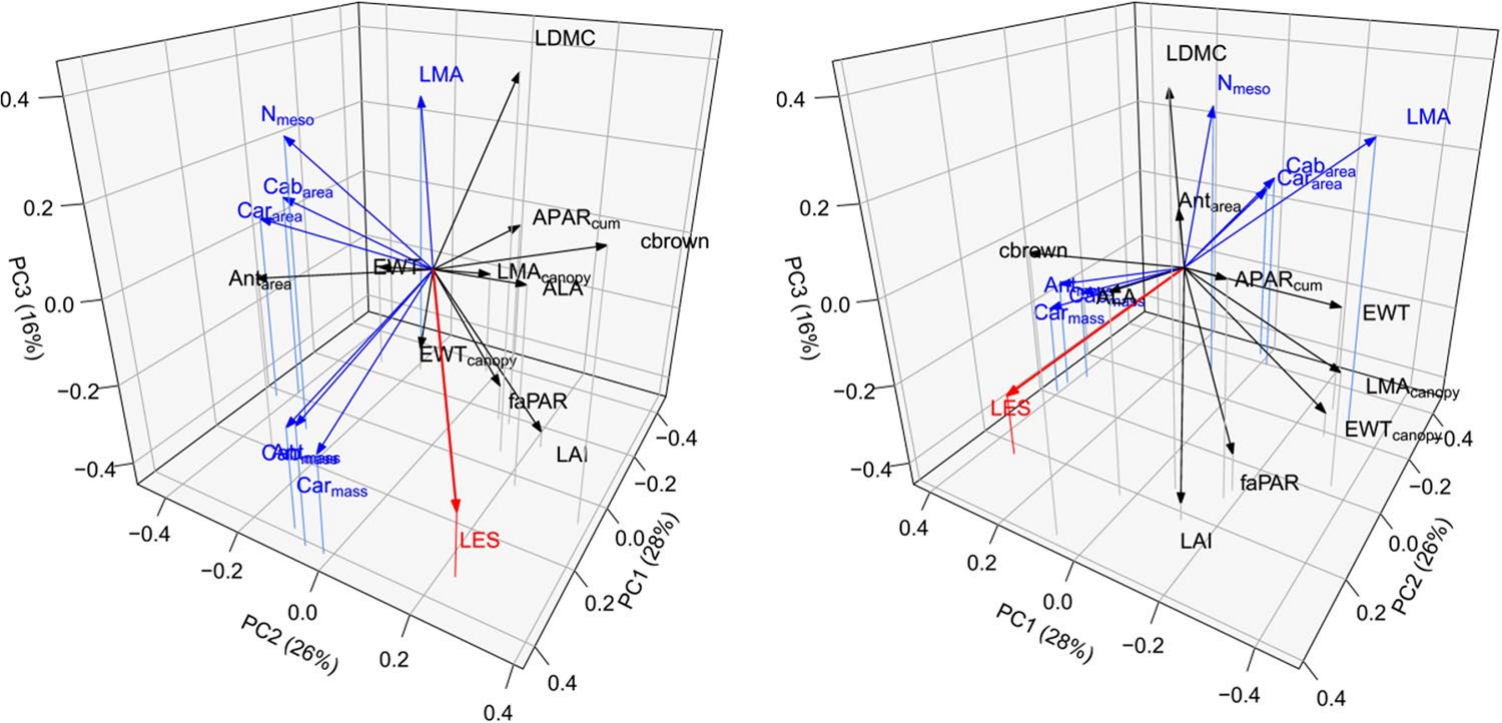
Two perspectives of the transformed trait space (principal component analysis) and relation to the Leaf Economic Spectrum (LES in red). Signifcant correlations between traits and the LES are highlighted in blue.

Which role do optically relevant traits play in relation to the LES? The set of optically relevant plant traits (compare Table 1) can be summarised using a three-dimensional feature space (Fig. 3), built by a principal component analysis, with component 1, 2 and 3 explaining 28%, 26% and 16% of the total variation, respectively. The LES was projected to this three-dimensional trait space to visualise relationships with optically relevant traits. As expected, LMA, as one of the original constituents of the LES, showed the highest correlation with the LES (−0.68 Pearson’s r). A lower yet positive correlation (p < 0.05) existed with pigment contents measured on a mass basis, i.e. Cab_mass_ (r = 0.42), Car_mass_ (r = 0.53), Ant_mass_ (r = 0.52), indicating decreasing pigment concentrations with increasing resource investments. Pigments measured on an area-basis also correlated significantly but negatively with the LES, i.e. Cab_area_ (r = −0.45), Car_area_ (r = −0.44), so that pigment contents predominantly increased with slow and conservative growth. Mesophyll thickness N_meso_ correlated negatively with the LES (r = −0.40), reflecting higher mesophyll thickness with increasing resource investments. Traits linked to leaf water content (EWT, EWT_canopy_, LDMC) and canopy structure (LAI, ALA, fAPAR, APAR_cum_) were not significantly correlated to the LES. A table with all correlations is provided in S5.

### Optically relevant traits versus CSR plant strategies

**Figure 4:**
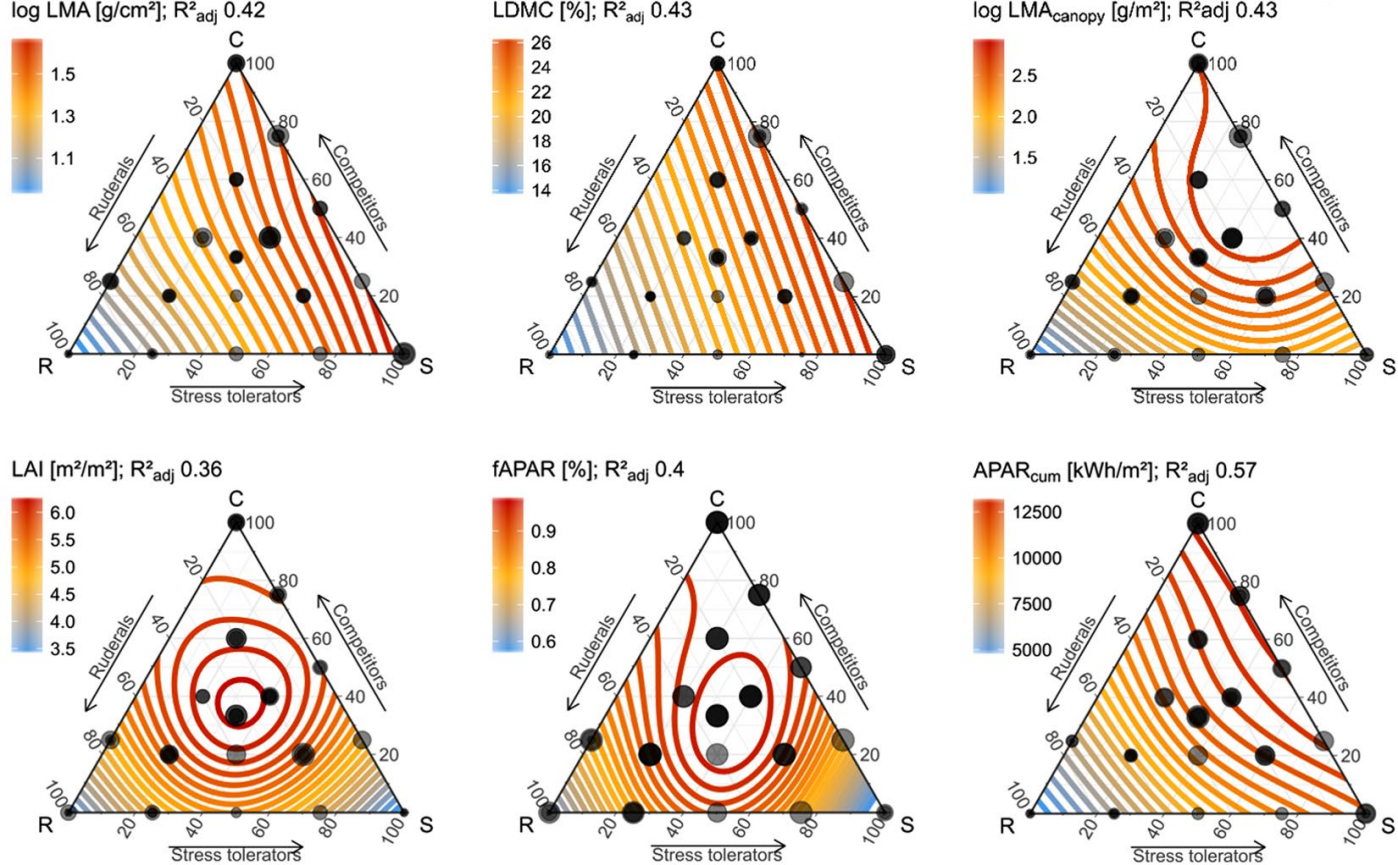
Distribution of optically relevant plant traits in the CSR- feature space of forbs and graminoids based on GAM extrapolations. Observations are displayed as transparent grey dots (partly overlapping) with a size proportional to the trait expression.

**Figure 5:**
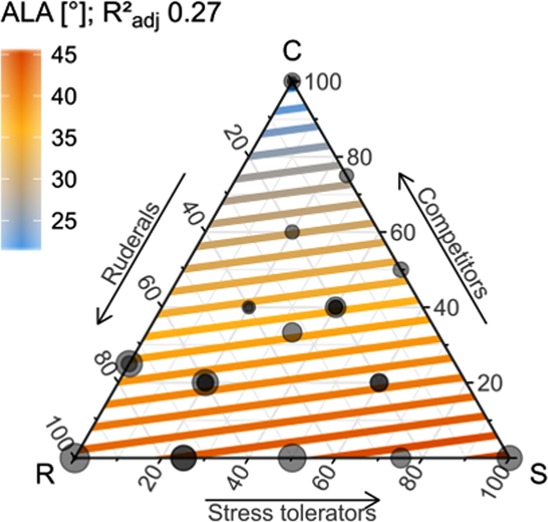
Distribution of average leaf angle (ALA) in the herbaceous CSR- feature space based on GAM extrapolations (details see caption Fig. 4).

**Figure 6:**
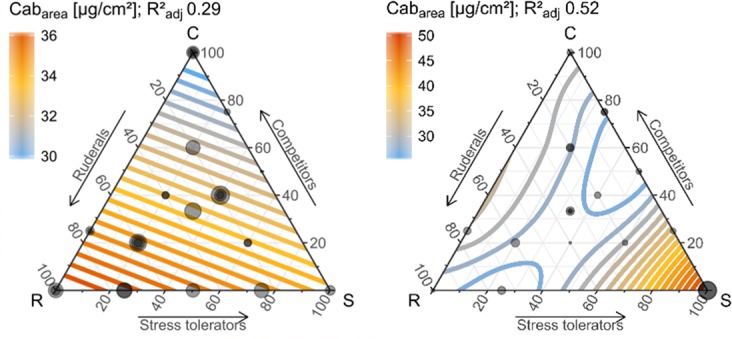
Distribution of Cab_area_ for herbaceous (left) and graminoid (right) CSR plant strategies based on GAM extrapolations (details see caption Fig. 4).

The distribution of optically relevant traits within the three-dimensional CSR scheme of plant strategies was assessed using thin plate regression splines^32^ and Generalized Additive Models (GAM)^33^. Neither EWT nor EWT_canopy_ correlated to plant strategies among forbs or graminoids. In contrast LAI, LMA, LMA_canopy_, LDMC, pigment_mass_, fAPAR and APAR_cum_ showed significant and consistent relationships across forbs and graminoids (Fig. 4). Pigment_area_, N_meso_, Cbrown and ALA exhibited no consistent relationship to CSR plant strategies across growth forms and thus related differently among forb and graminoid strategies (Figs 4–6). A table summarising the results for all traits is given in S6.

## Discussion

Vegetation canopies can be considered as solar power plants and various functions and traits are coordinated to ensure an efficient energy assimilation by adapting to environmental factors (e.g. nutrient availability, temperature) and biotic interactions (e.g. competition)^11,34^. Indeed, our results confirmed that plant functions and strategies are expressed through traits that directly affect or are directly related to optical processes in plant canopies and thus determine their reflectance. This relationship of ‘reflectance follows function’ firstly provides the physical basis for retrieving differences in plant functions by means of optical Earth observation data, and underlines the potential to track Earth’s functional diversity. Secondly, linking plant functions and strategies with radiative transfer provides a different and additional perspective on how environmental factors and biotic interactions shape plant functional diversity.

Why does reflectance of plant canopies follow function? Our results showed strong links between optically relevant plant traits and the two schemes used as baselines for plant functioning, i.e. the Leaf Economic Spectrum (LES) and CSR plant strategies. Originally the LES has been captured through leaf lifespan, LMA, photosynthetic capacity, dark respiration rate, nitrogen and phosphorus content^3^. Indeed, variation of observed LMA showed an strong correspondence to the LES, whereas we also found significant correlations with pigment_mass,_ and pigment_area_, both of which are directly linked to photosynthetic capacity and nitrogen content^35^. Our results further suggest that pigment content (pigment_area_) generally increases with leaf lifespan, whereas the concentration of pigments (pigment_mass_) decreases. Thus, plant investment in leaf tissue proportionally outweigh investment in leaf pigment, which can be explained as with increasing chlorophyll content light absorption follows a saturating curve, since chloroplasts become increasingly stacked in the palisade cells resulting in intraleaf shading^36–38^. Accordingly, plants with short leaf lifespan invest fewer pigment to optimise energy revenue. However, the LES reflects only one primary dimension of plant functioning, ranging from a quick to slow return on leaf resource investments. Accordingly, the canopy-structure-related traits (LAI, LMA_canopy_, ALA, *f*APAR or APAR_cum_) showed no significant correspondence to the LES, suggestingthat these traits are indeed related to other functional axes (Fig. 3). With the ‘global spectrum of plant form and function’ Diaz *et al*. have identified two major axes of plant functional convergence, with one axis reflecting leaf resource investments (LES) and the other axis reflecting plant and organ size-related traits. Accordingly, we expected that optically relevant traits integrating canopy properties are associated with the size-related axis. We actually observed such association between canopy-structure-related traits and the multidimensional CSR space, which characterises plant functioning in terms of competitive, stress tolerant and ruderal abilities at the whole plant level^31^. Accordingly, multiple traits that did not exhibit an association with the LES (e.g. LAI, LMA_canopy_, or *f*APAR), in turn showed a notable correspondence to the CSR space. Our results confirmed previous relationships between traits and the CSR space and exhibited gradients that have not been assessed before.

In agreement with pivotal formulations of the CSR scheme^29^ and the allocation by Hodgson *et al*.^39^, **LMA** was lowest for ruderal species and highest for stress tolerators, closely followed by competitors (Fig. 4). Leaf mass per canopy area, i.e. **LMA**_**canopy**_ (LMA • LAI), which was to our knowledge not compared to CSR strategies before, reflects total leaf carbon assimilation per canopy area. We found highest LMA_canopy_ for competitive species, followed by intermediate LMA_canopy_ for stress tolerators and intermediates and lowest values for ruderals. This gradient reflects the primary principles of the plant strategies^29^; stress tolerators feature a conservative growth with a long leaf lifespan resulting in a steady accumulation of dry matter in the canopy, whereas ruderals are adapted to disturbance events and thus have short lifecycles in which they accumulate few resources. Competitors feature both high productivity and a relatively long lifespan and therefore highest resource accumulation (LMA_canopy_).

Both leaf and canopy water content, i.e. **EWT** [g/cm²] and **EWT**_**canopy**_, did not show a clear relationship with plant strategies. In contrast **LDMC**, which is the ratio of leaf mass and leaf water content (LDMC = LMA/(LMA + EWT)), showed a clear coherence towards plant strategies and was therefore already used by Hodgson *et al*.^39^ to allocate CSR scores. This suggests that functional characteristics are expressed through the relative water concentration in leaf tissue, rather than the absolute leaf water content.

A more complex pattern was found for **LAI**, where intermediate species (CSR) have highest LAI values followed by competitors, and lowest LAI values correspond to high S and R scores. We thus expect that intermediate species (CSR) invest a large share of resources in foliage area, whereas competitors, ruderals and stress tolerators invest more resources towards their strategy-specific trait-expressions and functions. Competitors occur primarily in nutrient rich sites, where competition for sunlight is most pronounced and triggers increased height growth to overtop neighbouring individuals. Increased canopy height in turn requires additional resource investments in support tissues, e.g. in the stem for vertical plant growth itself as well as in enhanced leaf robustness (higher LMA), to compensate for increased exposure to wind (compare LMA gradient, Fig. 4)^40^. An increased LMA was also found for plants adapted to high light intensities (competitors) through increased palisade parenchyma to maximise photosynthetic capacity and thus quantum yield per unit leaf area and reduce potential light saturation^41,42^. This suggests that relative to intermediate strategies, competitors invest fewer resources in the development of total foliage area (LAI). Therefore, competition for sunlight might enforce a trade-off between the maximisation of height growth and light interception. This agrees with Porter *et al*.^43^, who have reported higher accumulation of leaves for shade tolerant species leaves with smaller canopy heights among tropical tree species.

**Leaf inclination (ALA)** did not show a trivial correspondence to the CSR spectrum, but differed between growth forms, reflecting generic differences in the canopy architecture of graminoids and forbs. Variation in leaf angles across graminoid strategies showed no explicit pattern. For forb strategies ALA increased from competitive forbs to stress tolerant and ruderal forbs (Fig. 5). This agrees with Hikosaka & Hirose^44^, who have simulated leaf angle distributions for plant canopies and found lower leaf angles with increasing competition. Competitive forbs, which aim to overtop and shade out the surrounding and competing plants, develop rather flat leaf angles to deplete or scatter most of the light before it is available for rivals. However, a horizontal leaf position requires increased support costs for petioles and branches, and is generally less efficient for light absorption as self-shading and light saturation increases^44,45^. Leaf angles hence increase with decreasing competition to scatter light between leaves and hence distribute light into the lower canopy^44,46^.

Similar to the LES-based analysis, the derived distributions of chlorophylls, carotenoids and anthocyanins across the CSR space are very alike (Figs 4, S6), since pigments are usually highly correlated in mature leaves^27^.Yet, the relationships differed greatly among pigments measured on an area basis (**pigment**_**mass**_) and measured on a mass basis (**pigment**_**area**_, Figs 6, S6). The relationship between pigment_area_ and CSR strategies further differed between forbs and graminoids (Fig. 6), which agrees with Tjoelker *et al*.^47^, who has found differences in leaf photosynthetic activity between grasses and forbs. For forbs ruderals and intermediates feature highest pigment_area_. Among graminoid strategies pigment_area_ showed a low and inconclusive variation across the CSR space apart from a strong increase for extremely stress tolerant graminoids (*Festuca ovina* and *Nardus stricta*).

Pigments normalised by mass (pigments_mass_) showed a very consistent gradient across growth forms (S8), which however almost exclusively mirrors the LMA gradient (r² of 0.74, 0.80, 0.85 for Cab_mass_, Car_mass_, Ant_mass_, respectively). This is further confirmed as the modelled pigment_mass_ values across the CSR-space were highly correlated with pigment_mass_ values based on a null-model, in which we sampled random pigments_area_ values that were subsequently divided by LMA and thus mass normalised (r² of 0.80, 0.91, 0.64 for Cab_mass_, Car_mass_, Ant_mass_, respectively). Accordingly, we found that pigments_mass_ indeed do not reflect pigment variation per se, but rather the LMA gradient, which varies in higher magnitudes than traits with photosynthetic function^48,49^. Likewise the strong correspondence between pigments_mass_ and the LES can largely be attributed by the high variation in LMA, as indicated by the null-model (S9). This indicates that the characterisation of plant canopies through pigments on a mass basis is greatly redundant with LMA and therefore appears to be not expedient, despite its frequent use in the remote sensing community^50,51^.

The distribution of simulated **fAPAR** across the CSR space showed a strong correspondence to LAI (Fig. 4), suggesting that variation in light harvesting is particularly determined by LAI (in line with^44,46,52^) and therefore highest for intermediate strategies followed by competitors. Yet, fAPAR solely represents the potential energy gain at a point in time and thereby does not consider phenological differences between plant strategies. Accordingly we modelled the accumulated photosynthetic active radiation **APAR**_**cum**_. This measure integrates fAPAR and the course of absorbed direct and diffuse radiation (assessed from HelioClim-3 archives^53^) during a plants phenological season (recorded for the cultivated plants). APAR_cum_ thus reflects the accumulated photosynthetic and carbon assimilation during a plant´s growth period^54^. APAR_cum_ showed a very consistent and clear pattern across growth forms. Corresponding to their short growth period ruderals featured the lowest APAR_cum_. Intermediate APAR_cum_ was found for stress tolerators, as these can compensate conditions that limit productivity through robustness and persistence, resulting in a comparably low but prolonged light harvesting. Highest APAR_cum_ was found for competitors, as competitive abilities require long-term investments (e.g., height growth) that are rewarded with long-term returns. These results thus showed that the phenology-dependent variation in energy acquisition directly reflects established plant strategies and functions. Moreover, the comparable strong relationship with APAR_cum_ emphasised that gradients in plant productivity are not fully reflected by a single biochemical or structural trait, but relate to the integrated response of pigments, LAI, ALA as well as phenology^22^. This particularly highlights the potential of multi-temporal Earth observation data to map functional gradients. The overall strong correlation between gradients derived from APAR_cum_ and LMA_canopy_ (r² = 0.88) underlines the plausibility of the models, as a large share of the absorbed energy is used for carbon assimilation in leaves^35^. The minor discrepancy between APAR_cum_ and LMA_canopy_ existed for competitors, where photosynthetic assimilation (APAR_cum_) is highest, but LMA_canopy_ showed a slight bias towards C-CSR, which could result from competitors investing a considerable part of their resources in height growth rather than total leaf tissue.

According to our results **Cbrown** and **N**_**meso**_ did not show a consistent relation with the CSR strategies across growth forms. Both traits only corresponded to CSR strategies among graminoids (S7). In agreement with Jacquemoud & Baret^55^ N_meso_ correlates with LMA. The distribution of Cbrown could not be explained in an ecological context. Overall, Cbrown and N_meso_ have a relatively low impact on canopy reflectance^56^ and do not greatly contribute to the spectral differentiation of variations in plant functioning^57^.

All trait measurements used in this study were retrieved from plants cultivated under optimal growth conditions. It can be expected that some traits more explicitly express their functional role under non-optimal environmental conditions. For instance, increased leaf anthocyanin content has been observed during pathogen infections^58^. Furthermore, a plant’s ability to cope with excess incident radiation can be expressed through developing ample leaf carotenoid content^27^.

A limitation of our study was the initial definition and selection of plant traits. Plant traits such as Leaf Area Index and the Average Leaf Angle are abstractions of complex canopy structural properties, and may not fully reflect subtle differences in plant functioning. Further errors are introduced by the trait measurementsand the analysis, such as GAM extrapolations, and the abstraction of plant functioning in the CSR model^29^ and the LES^3^. Yet, given that plant functioning and the radiative transfer in plant canopies are two complex fields on its own, empirically testing the links between these two realms requires a certain level of abstraction. Despite these uncertainties, our results showed unprecedented links between functioning and reflectance, hopefully triggering further advances towards this field using more sophisticated methods.

## Conclusion and Outlook

Optical remote sensing data is potentially highly informative to track Earth’s functional diversity. Yet, causal explanations on why plant functioning can be differentiated using canopy reflectance sensed by optical Earth observation data remain limited. Our findings demonstrate that bridging ecological theory and canopy reflectance through radiative transfer modelling enables us to identify causal links between canopy reflectance and plant functioning. These links suggest that canopy ‘reflectance follows function’, meaning that adaptations of plants to their environment are directly ‘reflected’ in their optical properties across the visible, near and short wave infrared wavelengths. More specifically plant functions and strategies are considerably expressed through multiple structural, physiological and phenological traits with relevance for canopy reflectance and thus optical Earth observation data. This opens up new opportunities for understanding plant functional changes in space and time. Increasing the dimension of relevant traits for an ecological system allows us to more precisely and completely understand and predict ecosystem dynamics and ecological processes such as community assembly^59^. Furthermore, trait ecology may lack a sufficient variety of traits to capture dissimilarities and explain competition among species^60^. As shown here optically relevant traits depict variations in multiple plant functions and can thus complement the suite of determinable proxies to describe spatial variation in plant functioning and community assembly. This is particularly emphasised as several optically relevant traits show comparable or even stronger correlations with CSR plant strategies (LAI, LMA_canopy_, APAR_cum_) than traits used originally to allocate the CSR space (e.g. LMA or LDMC^39^). Upcoming hyperspectral satellite missions such as EnMAP^61^ or HyspIRI^62^ will provide optical reflectance products that are sensitive to the traits considered in this study. Our results therefore encourage further research to deepen our understanding how plant functioning is expressed through optically relevant traits using more extensive trait data and further traits incorporated in more complex radiative transfer models (e.g., crown architecture), such as INFORM^63^ or FLIGHT^64^.

## Material and Methods

### Retrieval of the traits space implemented in PROSAIL

We derived the PROSAIL trait space from outdoor cultivated plants, including 45 forb and graminoid species covering the full range of the CSR spectrum (Table 2). We performed seed propagation in greenhouses and moved the plants outdoor for a week of acclimatisation once they were grown to an adequate size. Afterwards the plants were planted out in four repetitions in separate pots with a size of 0.4 m • 0.4 m and 30 l volume filled with a standardised substrate. Fewer repetitions had to be planted for species where seedling propagation was less successful. All pots were regularly fertilised, weeded and irrigated.

**Table 2:** List of all cultivated species.

Graminoids (n = 20)	Forbs (n = 25)
*Alopecurus geniculatus* (4, R/CSR); *Alopecurus pratensis* (4, C/CSR); *Anthoxanthum odoratum* (4, SR/CSR); *Apera spica-venti* (4, R/SR); *Agrostis capillaris* (4, CSR); *Arrhenatherum elatius* (4, C/CSR); *Brachypodium sylvaticum* (4, S/SC); *Bromus hordeaceus* (3, R/CR); *Calamagrostis epigejos* (4, C/SC); *Deschampsia cespitosa* (4, S/CSR);*Digitaria sanguinalis* (4, R/SR); *Festuca ovina* (4, S); *Holcus lanatus* (4, CSR); *Luzula multiflora* (4, S/CSR); *Molinia caerulea* (2, SC); *Nardus stricta* (4, S); *Phalaris arundinacea* (4, C); *Poa annua* (4, R); *Scirpus sylvaticus* (4, C/SC); *Trisetum flavescens* (4, CSR);	*Aegopodium podagraria* (4, CR/CSR); *Anthyllis vulneraria* (2, S/SR); *Arctium lappa* (4, C); *Centaurium erythraea* (4, SR); *Cirsium arvense* (4, C); *Cirsium acaule* (3, CS/CSR); *Digitalis purpurea* (4, CR/CSR); *Filipendula ulmaria* (1, C/SC); *Geum urbanum* (4, S/CSR); *Geranium pratense* (4, C/CSR); *Geranium robertianum* (R/CSR); *Plantago major* (4, R/CSR); *Clinopodium vulgare* (4, S/CSR); *Campanula rotundifolia* (4, S); *Lamium purpureum* (4, R); *Lapsana communis* (4, R/CR); *Medicago lupulina* (3, R/SR); *Origanum vulgare* (4, CS/CSR); *Pulicaria dysenterica* (4, CS); *Stellaria media* (4, R/CR); *Succisa pratensis* (3, CS/CSR); *Taraxacum spec*. (4, R/CSR); *Thlaspi arvense* (3, R); *Trifolium pratense* (4, CSR); *Urtica dioica* (4, C);

The number in parentheses indicates the number of repetitions per species followed by the allocated CSR strategy.

For each species we measured the considered traits on a weekly basis for each pot. We determined the species-specific trait expressions by averaging the measurements among pots and subsequently calculating the median for the whole season. We only considered measurements that were performed in non-senescent canopies of adult plants (here defined as plants with closed canopy).

In view of the envisaged amount of measurements per species traditional approaches for pigment retrieval such as the spectrophotometer method by Lichtenthaler^65^ was not feasible. Furthermore, N_meso_ and Cbrown are specific parameters of PROSPECT. We measured leaf chlorophyll content (Cab_area_), carotenoid content (Car_area_), anthocyanin content (Ant_area_), mesophyll structure coefficient (N_meso_) and brown pigment content (Cbrown) using leaf reflectance spectra and their inversion using the leaf radiative transfer model PROSPECT-D^27^. We acquired leaf spectra of 5 individual leaves per cultivated pot using an ASD FieldSpec III (ASD, Inc. Boulder, CO, USA) attached with a plant probe and leaf clip. If the area of a leaf was smaller than the opening of the plant probe (3.14 cm²) we seamlessly, and without overlap, placed the leaves side by side on an adhesive tape. The inversion of PROSPECT-D was based on a look-up-table approach and wavelets^66–68^. Further details on the inversion procedure and its validation are given in S1.

We estimated LAI using an Accu-PAR LP-80 ceptometer equipped with an external reference sensor to account for the current photosynthetic active radiation (PAR). For each pot we recorded and subsequently averaged 18 individual measurements.

To limited the destructice impact over time, we measured leaf mass per area (LMA) and equivalent water thickness (EWT) per species rather than per pot. Samples consistend of leaflets only without petioles and rachis. Fresh leaf mass of around 10 g of whole leaves per species was measured on site. Total leaf area of these leaf samples was retrieved using a flatbed scanner. The LMA [g/cm²] was derived by drying the sample material at 70 °C for at least 72 h. EWT [mg/cm²] was derived by subtracting LMA from leaf fresh mass per area.

The ALA was retrieved from leaf inclination distributions that we determined using levelled digital photograph and the procedure described by Ryu *et al*.^69^. For each species we measured not less than 50 angles of leaves parallel to the viewing direction. Leaf angle distributions and ALA were only retrieved once due to logistic constrains.

We deduced additional traits from the PROSAIL traits space to further exploit its information content: leaf dry matter content (LDMC = LMA/(LMA + EWT)); canopy leaf mass per area (LMA_canopy_ = LMA • LAI); canopy pigment content (pigment_canopy_ = pigment_area_• LAI); fraction of absorbed photosynthetic active radiation (*f*APAR) simulated using PROSAIL (details in S3)^25^; cumulative absorbed photosynthetic active radiation (APAR_cum_ in kWh/m²), which corresponds to the total absorbed energy within the growing season of each species. The APARcum was calculated as the product of *f*APAR, direct and diffuse irradiance averaged for April-October (data assessed from HelioClim-3 data^53^, details in S3) and the length of the growing season, (here defined as the observed number of weeks between maturity and senescence). A statistical summary of the trait space is given in S2.

### Linking the Leaf Economic Spectrum and optically relevant plant traits

Wright *et al*.^3^ determined the LES using the first component of a principal component transformation of six leaf traits, i.e. LMA, photosynthetic assimilation rate_mass_, leaf nitrogen_mass_, leaf phosphorus_mass_, dark respiration rate_mass_, and leaf lifespan. From those traits we only measured LMA (or SLA respectively) within the above described plant experiment. We therefore requested the remaining traits from the TRY-database, where sufficient data was available for 26 of the 45 species (see S4 for a list of the 26 species) and two further traits, leaf nitrogen_mass_, leaf phosphorus_mass_. We determined the LES for the 26 species using the log10 transformed expressions of these three traits and the loadings reported by Wright *et al*.^3^. The LES retrieved this way was compared to each of the optically relevant traits using Pearson´s correlation coefficient. Additionally, the relationship among the different optically relevant traits and their relation to the LES was visualised by means of a principal component analysis (PCA). Therefore, we built a PCA of the PROSAIL trait space on which the LES was projected using the function (‘envfit’ of the vegan package). Prior to the PCA the PROSAIL traits were centered and scaled.

### Linking CSR plant strategies and optically relevant plant traits

The position of a species in the CSR space is defined by three axes expressing competitive, stress tolerant and ruderal abilities (scores). We used the CSR scores provided by Hodgson *et al*.^39^, who allocated CSR strategies for a multitude of European plant species using trait expressions of canopy height, LDMC, flowering period, flowering start, lateral spread, LMA and specific leaf area (Table 2). For some species we adopted the allocation from the BiolFlor database^70^ and expert knowledge.

We assessed the relationship between each PROSAIL trait and the CSR space using Generalized Additive Models (GAM)^33^ and thin plate regression splines^32^. As input for the GAM we used the first two PCA components (cumulative variance 97%) instead of the raw CSR scores to facilitate the interpretability of the results. The results were visualised in ternary plots (R-package ‘ggtern’)^71^.

## Supplementary information

Supplementary Informaton
