# Supplementary material for: Radiative transfer modelling reveals why canopy reflectance follows function
Source: Sci Rep. 2019 Apr 25;9:6541. doi: 10.1038/s41598-019-43011-1 (PMC6484002; doi:10.1038/s41598-019-43011-1)
Supplement: Supplementary file 1 — Supplementary Informaton [file 41598_2019_43011_MOESM1_ESM.pdf]

Supplementary information:

Radiative transfer modelling reveals why canopy reflectance follows function

Teja Kattenborn*, Sebastian Schmidtlein

Institute of Geography and Geoecology (IFGG), Karlsruhe Institute of Technology (KIT), Kaiserstr. 12, 76131 Karlsruhe, Germany

Supplementary information 1: Inversion of PROSPECT-D for the retrieval of $chlorophyll_{area}$, $carotenoid_{area}$, $anthocyannin_{area}$, Cbrown and N_{meso}

As a compromise between robustness and computation speed, we selected a look-up-table size of 100.000. The range of each trait for the generation of the LUT is shown in Table S1-1. We simulated spectra using PROSPECT-D [1]. Previous studies have demonstrated that wavelet transformations improve the parameter retrieval of RTM inversions [2,3,4,5]. Wavelet transformation decompose the hyperspectral signature into frequency components at different spectral scales, which facilitates the retrieval of the spectral features. Thus, we identified the simulated LUT spectra with the closest correspondence to the ASD spectra using continuous wavelet transformations. The wavelets were calculated using the R-package 'wmtsa' (settings: number of scales, i.e. wavelets = 8, scale range = 1 to 350). We excluded the wavelets of scale 1 to 3, as these primarily represent noise and artefacts and only used the wavelets 4 to 8 in the analysis. To identify the closest match between the wavelet transformations of LUT and ASD spectra we used the RMSE as cost function. We derived the final estimates for each trait by selecting the 20 LUT entries which resulted in the smallest RMSE. As proposed by Vohland et al. [6] we weighted and subsequently averaged the trait values of these LUT entries according to their RMSE value.

We validated the above described inversion procedure using the ANGERS leaf optical properties database, which was acquired in 2003 at INRA, France [7]. The data base contains 276 leaf reflectance spectra (400nm – 2450nm) for 43 different species as well as reference values of inter alia chlorophyll (a+b) content [µg/cm²], carotenoid content [µg/cm²] and N_{meso}. After applying the inversion on the leaf spectra the accuracy of the trait was assessed using the R^2 and NRMSE (see Tab. S1-2). A validation of the estimated brown pigment content was not possible, since the LOPEX dataset does not contain respective reference values. However, the relatively accurate retrieval of chlorophyll content, carotenoid content and the mesophyll structure coefficient indicate the overall robustness of the inversion procedure.

Table S1-1: The range of each parameter for the inversion of leaf spectra using PROSPECT-D

PROSPECT-D parameter/ Trait	Abbrev.	Min	Max
Chlorophyll content [µg cm^{-2}]	Cab_{area}	1	110
Carotenoid content [µg cm^{-2}]	Car_{area}	1	26
Anthocyannin content [µg cm^{-2}]	Ant_{area}	0.1	4
Mesophyll structure coefficient [ ]	N_{meso}	0.8	2.8
Leaf mass per area [g cm^{-2}]	LMA	0.0015	0.033
Water content [g cm^{-2}]	EWT	0.004	0.055
Brown pigment content [ ]	Cbrown	0.0	0.4

Table S1-2: Validation of the PROSPECT inversion procedure for $chlorophyll_{area}$, $carotenoid_{area}$ and N_{meso} coefficient using the ANGERS leaf optical properties database

Trait	R^2	NRMSE [%]
Chlorophyll content [µg cm^{-2}],	0.91	7.91
Carotenoid content [µg cm^{-2}],	0.66	15.1
Mesophyll structure coefficient [ ]	0.77	11.6

Supplementary information 2: Statistical summary of the measured traits implemented in PROSAIL and derivates thereof

Table S2-1. Statistical summary of the measured traits implemented in PROSAIL and derivates thereof.

Trait [unit]	min	max	mean	median	sd
**ALA [°]**	11.325	70.115	42.847	43.252	15.86
**LAI [m^2 m^{-2}]**	1.096	8.758	5.311	5.428	1.79
**LMA [g cm^{-2}]**	1.759	8.923	4.319	4.097	1.658
**EWT [g cm^{-2}]**	0.009	0.046	0.016	0.014	0.007
**LDMC [%]**	0.102	0.379	0.216	0.217	0.061
**Cab_{area} [ug cm^{-2}]**	19.73	54.505	32.349	30.376	8.022
**Car_{area} [ug cm^{-2}]**	6.695	12.188	9.256	8.944	1.39
**Ant_{area} [ug cm^{-2}]**	0.716	2.188	1.241	1.249	0.325
**$logLMA_{canopy}$ [g m^{-2}]**	40.023	510.496	220.278	200.566	100.776
**EWT_{canopy} [g m^{-2}]**	0.012	0.239	0.082	0.072	0.044
**Cab_{mass} [ug g^{-1}]**	3.854	28.041	9.493	8.111	4.904
**Car_{mass} [ug g^{-1}]**	1.024	7.12	2.716	2.432	1.279
**Ant_{mass} [ug g^{-1}]**	0.123	1.197	0.371	0.353	0.209
***f*APAR [%]**	0.595	0.967	0.933	0.956	0.066
**$APAR_{cum}$ [kWh m^{-2}]**	2.34	23.802	14.997	15.822	5.446
**Cbrown [ ]**	0.047	0.137	0.107	0.109	0.021
**N_{meso} [ ]**	1.107	1.723	1.343	1.302	0.178

Supplementary information 3: Derivation of fAPAR and $APAR_{cum}$

For each species we simulated fAPAR using the radiative transfer model PROSAIL parametrized with the retrieved trait expressions. fAPAR was calculated based on the method provided in Verhoef & Bach [8] and the following formula:

$$\mathrm{fAPAR} = \frac{1}{24}\sum_{\theta=1}^{24} \frac{\sum_{400}^{700} \alpha_{s_\theta} * E_{sun_\theta} + \alpha_{d_\theta} * E_{sky_\theta}}{\sum_{400}^{700} E_{sun_\theta} + E_{sky_\theta}} \qquad \text{eqn S3-1}$$

$$\alpha_s = 1 - r_{sd} - \tau_{sd} - \tau_{ss} \qquad \text{eqn S3-2}$$

$$\alpha_d = 1 - r_{sd} - \tau_{dd} \qquad \text{eqn S3-3}$$

where E_{sun} is the solar irradiance at ground level, E_{sky} is the sky irradiance at ground level, r_{sd} is the soil surface reflectance, τ_{sd} is the directional-hemispherical transmittance for solar flux, τ_{ss} is the direct transmittance for solar flux and τ_{dd} is the bi-hemispherical transmittance. For simplicity fAPAR was integrated for the course of a day in central Germany (01st August, Lat. 48°, Long. 8°).

$APAR_{cum}$ was derived from fAPAR, photosynthetic active radiation (PAR) [kWh/m^2] and the number of growing days for each cultivated species. Hourly PAR values were derived from the default radiation albedos (E_{sun} and E_{sky}) in PROSAIL (400-700 nm) scaled with averaged direct and diffuse radiation for April-October (2016, Lat. 48°, Long. 8°) assessed from HelioClim-3 archives [9].

$$\mathrm{APAR_{cum}} = d_{growth} * \sum_{\theta=1}^{24} \mathrm{fAPAR}_\theta * (\mathrm{PAR}_{sun_\theta} + \mathrm{PAR}_{sky_\theta}) \qquad \text{eqn S3-4}$$

where d_{growth} is the number of growing days, θ is the sun angle at a given hour of the day, $\mathrm{PAR}_{sun_\theta}$ and $\mathrm{PAR}_{sky_\theta}$ are the direct and diffuse photosynthetic active radiation, respectively.

Supplementary information 4: Species considered in the LES analysis

Aegopodium podagraria, Anthyllis vulneraria, Anthoxanthum odoratum, Alopecurus pratensis, Arctium lappa, Arrhenatherum elatius, Calamagrostis epigejos, Campanula rotundifolia, Centaurium erythraea, Cirsium acaule, Cirsium arvense, Geranium pretense, Geranium robertianum, Festuca ovina, Holcus lanatus, Molinia caerulea, Nardus stricta, Phalaris arundinaceae, Plantago major, Poa annua, Trifolium pratense, Trisetum flavescens, Stellaria media, Succisa pratensis, Urtica dioica

Supplementary information 5: Correlation of optically relevant plant traits and the Leaf Economic Spectrum

Table S5-1. The correlation (pearson's r) between each optically relevant trait and the Leaf Economic Spectrum

Trait	r trait ~ LES	p-value
**ALA**	-0.21	0.308
**LAI**	0.07	0.752
**LMA**	-0.68	0.000
**EWT**	-0.31	0.131
**LDMC**	-0.28	0.178
**Cab_{area}**	-0.45	0.023
**Car_{area}**	-0.44	0.028
**Ant_{area}**	0.00	0.992
**LMA_{canopy}**	-0.36	0.079
**EWT_{canopy}**	-0.22	0.293
**Cab_{mass}**	0.42	0.037
**Car_{mass}**	0.53	0.006
**Ant_{mass}**	0.52	0.008
***f*APAR**	0.04	0.841
**$APAR_{cum}$**	-0.23	0.258
**Cbrown**	0.32	0.114
**N_{meso}**	-0.4	0.046

Supplementary information 6: Relationship of optically relevant traits and CSR plant strategies

Tab. S6-1: Adjusted R^2 and p-values of the relationship between the CSR feature space and plant traits derived using generalized additive models for all species (n=45), graminoids (n=19) and forbs (n=26).

Trait	R^2_{adj} all	p-all	R^2_{adj} grass	p-grass	R^2_{adj} forb	p-forb
**ALA [°]**	-0.06	0.891	-0.34	0.844	**0.33**	0.216
**LAI [m^2 m^{-2}]**	**0.36**	0.001	**0.6**	0.055	**0.45**	0.018
**LMA [g cm^{-2}]**	**0.42**	0	**0.73**	0.008	**0.48**	0.013
**EWT [mg cm^{-2}]**	-0.14	0.928	0.01	0.567	-0.12	0.962
**LDMC [g g^{-1}]**	**0.43**	0.003	**0.34**	0.025	0.4	0.003
**Cab_{area} [ug cm^{-2}]**	0.2	0.088	**0.52**	0.006	**0.29**	0.088
**Car_{area} [ug cm^{-2}]**	0.11	0.159	**0.41**	0.068	**0.22**	0.147
**Ant_{area} [ug cm^{-2}]**	0.19	0.049	**0.4**	0.092	**0.22**	0.146
**LMA_{canopy} [g m^{-2}]**	**0.43**	0	**0.39**	0.018	**0.4**	0.031
**EWT_{canopy} [mg m^{-2}]**	0.02	0.401	0.07	0.433	-0.06	0.69
**Cab_{mass} [ug g^{-1}]**	**0.57**	0	**0.47**	0.074	**0.67**	0
**Car_{mass} [ug g^{-1}]**	**0.55**	0	**0.53**	0.045	**0.61**	0.002
**Ant_{mass} [ug g^{-1}]**	**0.37**	0.003	**0.41**	0.134	**0.44**	0.031
**Cbrown [ ]**	-0.09	0.777	**0.46**	0.107	-0.08	0.791
**N_{meso} [ ]**	0.08	0.101	**0.29**	0.045	-0.09	0.919
***f*APAR [%]**	**0.37**	0.002	**0.68**	0.017	**0.63**	0.001
**$aPAR_{cum}$ [kWh m^{-2}]**	**0.57**	0	**0.57**	0.001	**0.57**	0

Supplementary information 7: N_{meso} and Cbrown gradients across graminoid growth forms

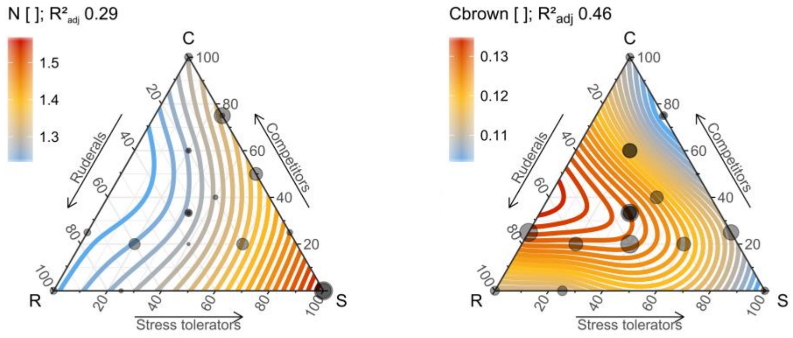

Fig. S7-1. Distribution of mesophyll thickness (N_{meso}) and Brown pigment content (Cbrown) across graminoid CSR strategies based on GAM extrapolations (details see caption Fig. 4).

Supplementary information 8: Null model of pigment_{mass} vs CSR plant strategies

The information content of pigment_{mass} was assessed using a null model that involved random sampling of pigment_{area} values within the range of the in-situ measurements, which were subsequently mass-normalized (divided by LMA). As shown below, the resulting artificial pigment_{mass} values and their relation to CSR plant strategies show a great correspondence to the actual values of pigment_{mass}, indicating that pigment_{mass} primarily reflects variation in LMA.

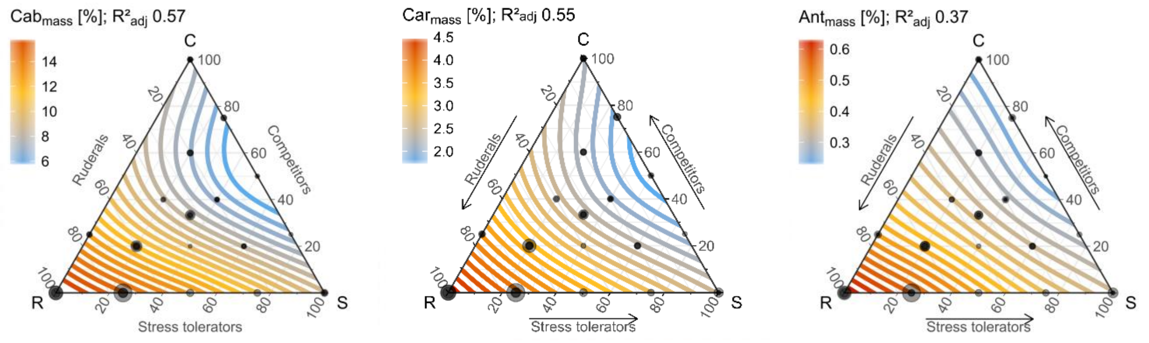

Fig. S8-1. Distribution of pigments_{mass} across CSR strategies (graminoids and forbs) based on GAM extrapolations (details see caption Fig. 4).

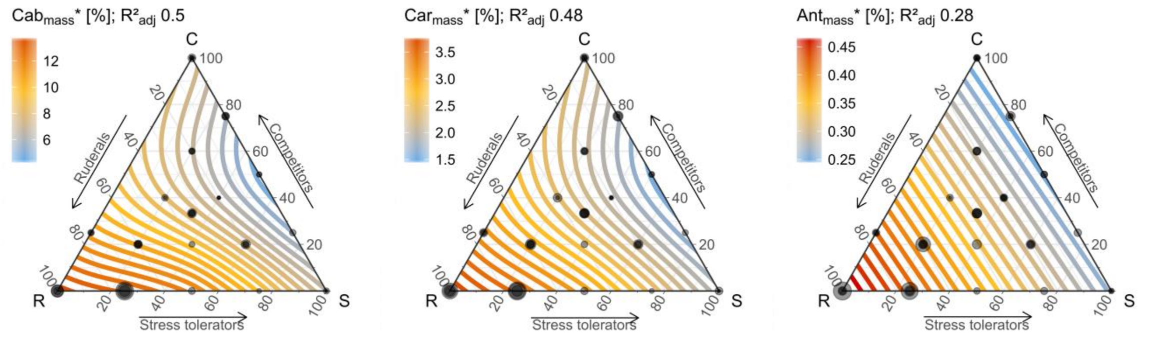

Fig. S8-2. Distribution of pigments_{mass} derived from the null model across CSR strategies (graminoids and forbs) based on GAM extrapolations (details see caption Fig. 4).

Supplementary information 9: Null model of pigment$_{mass}$ vs Leaf Economic Spectrum

The information content of pigment$_{mass}$ was assessed using a null model that involved random sampling of pigment$_{area}$ values within the range of the in-situ measurements, which were subsequently mass-normalized (divided by LMA). As shown below the resulting pigment$_{mass}$ values and its relation to the LES show a clear correspondence to the actual values of pigment$_{mass}$, indicating that pigment$_{mass}$ primarily reflects variation in LMA.

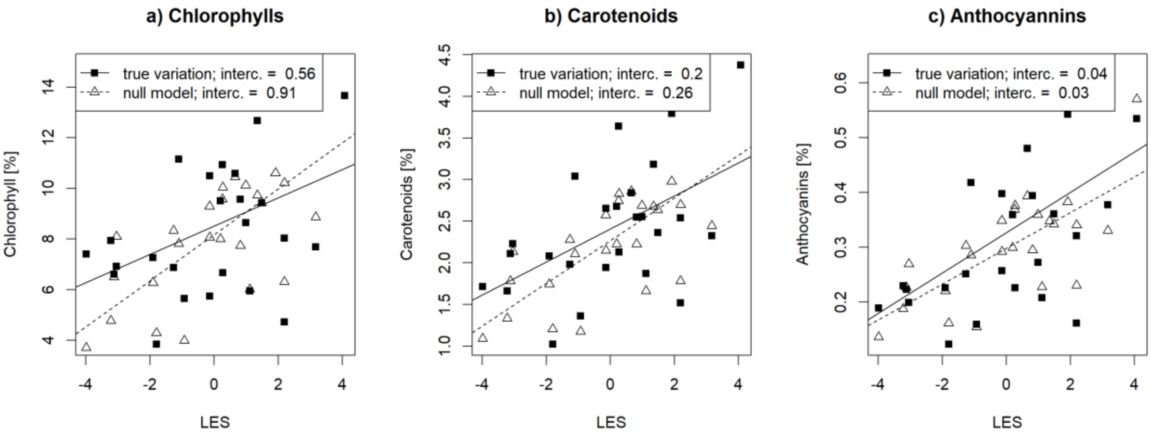

Fig. S9-1. The relation of original and artificial (null-model) pigment$_{mass}$ values towards the Leaf Economic Spectrum.

Supplementary information 10: Data used for deriving the Leaf economic spectrum made available through the TRY-database

Adler, P. B., Salguero-Gómez, R., Compagnoni, A., Hsu, J. S., Ray-Mukherjee, J., Mbeau-Ache, C., & Franco, M. (2014). Functional traits explain variation in plant life history strategies. Proceedings of the National Academy of Sciences, 111(2), 740-745.

Bakker, C., P. M. Van Bodegom, H. J. M. Nelissen, W. H. O. Ernst, and R. Aerts (2006). Plant responses to rising water tables and nutrient management in calcareous dune slacks. Plant Ecology 185, 19-28.

Bakker, C., J. Rodenburg, and P. Bodegom (2005). Effects of Ca- and Fe-rich seepage on P availability and plant performance in calcareous dune soils. Plant and Soil 275, 111-122.

Burrascano, S., Copiz, R., Del Vico, E., Fagiani, S., Giarrizzo, E., Mei, M., ... & Blasi, C. (2015). Wild boar rooting intensity determines shifts in understorey composition and functional traits. Community ecology, 16(2), 244-253.

Campetella, G; Botta-Duk\xe1t, Z; Wellstein, C; Canullo, R; Gatto, S; Chelli, S; Mucina, L; Bartha, S (2011). Patterns of plant trait-environment relationships along a forest succession chronosequence. Agriculture, Ecosystems & Environment, 145(1), 38-48. doi:10.1016/j.agee.2011.06.025

Cerabolini B., Pierce S., Luzzaro A., Ossola A. (2010). Species evenness affects ecosystem processes in situ via diversity in the adaptive strategies of dominant species. Plant Ecology, 207(2), 333-345

Cerabolini B.E.L., Brusa G., Ceriani R.M., De Andreis R., Luzzaro A. & Pierce S. (2010). Can CSR

De Vries F., Bardgett R.D. (2016). Plant community controls on short-term ecosystem nitrogen retention. New Phytologist. doi: 10.1111/nph.13832´.

Fitter, A. H. and H. J. Peat (1994). The Ecological Flora Database. Journal of Ecology 82, 415-425.

Han, W. X., J. Y. Fang, D. L. Guo, and Y. Zhang (2005). Leaf nitrogen and phosphorus stoichiometry across 753 terrestrial plant species in China. New Phytologist 168, 377-385.

Hickler, T. (1999). Plant functional types and community characteristics along environmental gradients on Oland's Great Alvar (Sweden). Sweden: University of Lund.

Everwand G, Fry, EL, Eggers T, Manning P (2014). Seasonal variation in the relationship between plant traits and grassland carbon and water fluxes. Ecosystems 17, 1095-1108.

Fry, E.L., Power, S.A. Manning, P. (2014). Trait based classification and manipulation of functional groups in biodiversity-ecosystem function experiments. Journal of Vegetation Science, 25, 248-261.

Garnier, E., S. Lavorel, P. Ansquer, H. Castro, P. Cruz, J. Dolezal, O. Eriksson, C. Fortunel, H. Freitas, C. Golodets, K. Grigulis, C. Jouany, E. Kazakou, J. Kigel, M. Kleyer, V. Lehsten, J. Leps, T. Meier, R. Pakeman, M. Papadimitriou, V. P. Papanastasis, H. Quested, F. Quetier, M. Robson, C. Roumet, G. Rusch, C. Skarpe, M. Sternberg, J.-P. Theau, A. Thebault, D. Vile, and M. P. Zarovali (2007). Assessing the effects of land-use change on plant traits, communities and ecosystem functioning in grasslands: A standardized methodology and lessons from an application to 11 European sites. Annals of Botany 99, 967-985.

Kattge, J., W. Knorr, T. Raddatz, and C. Wirth (2009). Quantifying photosynthetic capacity and its relationship to leaf nitrogen content for global-scale terrestrial biosphere models. Global Change Biology 15, 976-991.

Kerkhoff, A. J., W. F. Fagan, J. J. Elser, and B. J. Enquist (2006). Phylogenetic and growth form variation in the scaling of nitrogen and phosphorus in the seed plants. American Naturalist 168, 103-122.

Kleyer, M., R. M. Bekker, I. C. Knevel, J. P. Bakker, K. Thompson, M. Sonnenschein, P. Poschlod, J. M. van Groenendael, L. Klimes, J. Klimesova, S. Klotz, G. M. Rusch, Hermy, M. , D. Adriaens, G. Boedeltje, B. Bossuyt, A. Dannemann, P. Endels, L. G\xf6tzenberger, J. G. Hodgson, A.-K. Jackel, I. K\xfchn, D. Kunzmann, W. A. Ozinga, C. R\xf6mermann, M. Stadler, J. Schlegelmilch, H. J. Steendam, O. Tackenberg, B. Wilmann, J. H. C. Cornelissen, O. Eriksson, E. Garnier, and B. Peco (2008). The LEDA Traitbase: a database of life-history traits of the Northwest European flora. Journal of Ecology 96, 1266-1274.

Milla, R., & Reich, P. B. (2011). Multi-trait interactions, not phylogeny, fine-tune leaf size reduction with increasing altitude. Annals of Botany, 107(3), 455-465.

Meziane, D. and B. Shipleyn (1999). Interacting determinants of specific leaf area in 22 herbaceous species: effects of irradiance and nutrient availability. Plant Cell and Environment 22, 447-459.

Kichenin, E., Wardle, D. A., Peltzer, D. A., Morse, C. W., & Freschet, G. T. (2013). Contrasting effects of plant inter-and intraspecific variation on community-level trait measures along an environmental gradient. Functional Ecology, 27(5), 1254-1261.

Komac B, C Pladevall, M Domenech and R Fanlo (2014). Functional diversity and grazing intensity in sub-alpine and alpine grasslands in Andorra. Applied Vegetation Science Doi: 10.1111/avsc.12119

Maire, V., Wright, I. J., Prentice, I. C., Batjes, N. H., Bhaskar, R., van Bodegom, P. M., ... & Reich, P. B. (2015). Global effects of soil and climate on leaf photosynthetic traits and rates. Global Ecology and Biogeography, 24(6), 706-717.

Lhotsky, B., Csecserits, A., Kovács, B., & Botta-Dukát, Z. (2016). New plant trait records of the Hungarian flora. Acta Botanica Hungarica, 58(3-4), 397-400.

Ordonez, J. C., P. M. van Bodegom, J. P. M. Witte, R. P. Bartholomeus, J. R. van Hal, and R. Aerts. (2010). Plant Strategies in Relation to Resource Supply in Mesic to Wet Environments: Does Theory Mirror Nature? American Naturalist 175, 225-239.

Peco B., de Pablos I., Traba J. , & Levassor C (2005). The effect of grazing abandonment on species composition and functional traits: the case of dehesa Basic and Applied Ecology, 6(2), 175-183

Pierce S., Ceriani R.M., De Andreis R., Luzzaro A. & Cerabolini B. (2007). The leaf economics spectrum of Poaceae reflects variation in survival strategies. Plant Biosystems 141(3), 337-343.

Pierce S., Luzzaro A., Caccianiga M., Ceriani R.M. & Cerabolini B. (2007). Disturbance is the principal ?-scale filter determining niche differentiation, coexistence and biodiversity in an alpine community. Journal of Ecology 95, 698-706.

Pierce S., Brusa G., Vagge I., Cerabolini B.E.L. (2013) Allocating CSR plant functional types: the use of leaf economics and size traits to classify woody and herbaceous vascular plants. Functional Ecology, 27(4), 1002-1010

Prentice, I. C., Meng, T., Wang, H., Harrison, S. P., Ni, J., & Wang, G. (2011). Evidence of a universal scaling relationship for leaf CO2 drawdown along an aridity gradient. New Phytologist, 190(1), 169-180.

Reich, P. B., J. Oleksyn, and I. J. Wright. (2009). Leaf phosphorus influences the photosynthesis-nitrogen relation: a cross-biome analysis of 314 species. Oecologia 160, 207-212.

Schroeder-Georgi, T., Wirth, C., Nadrowski, K., Meyer, S. T., Mommer, L., & Weigelt, A. (2016). From pots to plots: hierarchical trait-based prediction of plant performance in a mesic grassland. Journal of Ecology, 104(1), 206-218.

Shipley, B. and M. J. Lechowicz. (2000). The functional co-ordination of leaf morphology, nitrogen concentration, and gas exchange in 40 wetland species. Ecoscience 7, 183-194.

Shipley, B. and T. T. Vu. (2002). Dry matter content as a measure of dry matter concentration in plants and their parts. New Phytologist 153, 359-364.

Shipley, B. (2002). Trade-offs between net assimilation rate and specific leaf area in determining relative growth rate: relationship with daily irradiance. Functional ecology, 16(5), 682-689.

Shipley, B. (1995). Structured Interspecific Determinants of Specific Leaf-Area in 34 Species of Herbaceous Angiosperms. Functional Ecology 9, 312-319.

Sandel, B., Corbin, J. D., & Krupa, M. (2011). Using plant functional traits to guide restoration: a case study in California coastal grassland. Ecosphere, 2(2), 1-16.

Siefert, A. (2012). Spatial patterns of functional divergence in old-field plant communities. Oikos 121,

Siefert, A., Fridley, J.D., and Ritchie, M.E. (2014). Community functional responses to soil and climate at multiple spatial scales: when does intraspecific variation matter? PLOS ONE 9

Takkis, K. (2014). Changes in plant species richness and population performance in response to habitat loss and fragmentation. (Dissertation). University Tartuenis, 2014-04-07. Available from: http://hdl.handle.net/10062/39546

Vergutz, L., S. Manzoni, A. Porporato, R.F. Novais, and R.B. Jackson. (2012). A Global Database of Carbon and Nutrient Concentrations of Green and Senesced Leaves. Data set. Available on-line [http://daac.ornl.gov] from Oak Ridge National Laboratory Distributed Active Archive Center, Oak Ridge, Tennessee, U.S.A. http://dx.doi.org/10.3334/ORNLDAAC/1106

Vohland, M., Mader, S., & Dorigo, W. (2010). Applying different inversion techniques to retrieve stand variables of summer barley with PROSPECT+ SAIL. International Journal of Applied Earth Observation and Geoinformation, 12(2), 71-80.

Han, W., Chen, Y., Zhao, F. J., Tang, L., Jiang, R., & Zhang, F. (2012). Floral, climatic and soil pH controls on leaf ash content in China's terrestrial plants. Global Ecology and Biogeography, 21(3), 376-382.

Wright, I. J., P. B. Reich, M. Westoby, D. D. Ackerly, Z. Baruch, F. Bongers, J. Cavender-Bares, T. Chapin, J. H. C. Cornelissen, M. Diemer, J. Flexas, E. Garnier, P. K. Groom, J. Gulias, K. Hikosaka, B. B. Lamont, T. Lee, W. Lee, C. Lusk, J. J. Midgley, M. L. Navas, U. Niinemets, J. Oleksyn, N. Osada, H. Poorter, P. Poot, L. Prior, V. I. Pyankov, C. Roumet, S. C. Thomas, M. G. Tjoelker, E. J. Veneklaas, and R. Villar. (2004). The worldwide leaf economics spectrum. Nature 428, 821-827.

Willis, C. G., M. Halina, C. Lehman, P. B. Reich, A. Keen, S. McCarthy, and J. Cavender-Bares. (2010). Phylogenetic community structure in Minnesota oak savanna is influenced by spatial extent and environmental variation. Ecography 33, 565-577.

References

1. Féret, J.-B., Gitelson, A. A., Noble, S. D., & Jacquemoud, S. (2017). PROSPECT-D: Towards modeling leaf optical properties through a complete lifecycle. Remote Sensing of Environment, 193, 204–215.

2. Blackburn, G. A. (2007). Wavelet decomposition of hyperspectral data: a novel approach to quantifying pigment concentrations in vegetation. International Journal of Remote Sensing, 28(12), 2831-2855.

3. Blackburn, G. A., & Ferwerda, J. G. (2008). Retrieval of chlorophyll concentration from leaf reflectance spectra using wavelet analysis. Remote Sensing of Environment, 112(4), 1614-1632.

4. Cheng, T., Rivard, B., & Sanchez-Azofeifa, A. (2011). Spectroscopic determination of leaf water content using continuous wavelet analysis. Remote Sensing of Environment, 115(2), 659–670

5. Kattenborn, T., Fassnacht, F. E., Pierce, S., Lopatin, J., Grime, J. P., & Schmidtlein, S. (2017). Linking plant strategies and plant traits derived by radiative transfer modelling. Journal of vegetation science, 28(4), 717-727.

6. Vohland, M., Mader, S., & Dorigo, W. (2010). Applying different inversion techniques to retrieve stand variables of summer barley with PROSPECT+ SAIL. International Journal of Applied Earth Observation and Geoinformation, 12(2), 71-80.

7. Jacquemound, S., Bidel, L., Francois, C., & Pavan, G. (2003). ANGERS Leaf Optical Properties Database (2003). Data Set. Available online: http://ecosis.org (accessed on 2 February 2019).

8. Verhoef, W., & Bach, H. (2007). Coupled soil–leaf-canopy and atmosphere radiative transfer modeling to simulate hyperspectral multi-angular surface reflectance and TOA radiance data. Remote Sensing of Environment, 109(2), 166-182.

9. Espinar, B., Blanc, P., Wald, L., Gschwind, B., Ménard, L., Wey, E., ... & Saboret, L. (2012). HelioClim-3: a near-real time and long-term surface solar irradiance database. Workshop on" Remote Sensing Measurements for Renewable Energy".
